# The Administrative Advance

**Published:** 1890-10-11

**Authors:** 


### The Hospital

A Weekly Journal of

**Science, Medicine, Nursing, and Philanthropy.**

*For Hospitals, Asylums, Medical Practitioners, Students, Nurses, Families, Parishes, Congregations, and the Charitable Public.*

Vol. IX.—No. 211.] [October 11, 1890.

### The Administrative Advance.

This is an age of experts. If anything has to be done by anyone who desires to be even with the times, he inquires who is regarded as the chief authority on the subject in question, and generally places himself unreservedly in the expert's hands. Experts, like other people, have their faults. A man who has devoted many years of his life to one subject, and who has worked at it with zeal and enthusiasm, naturally acquires a fulness of knowledge not possessed by others. After a time he finds, as he comes into contact with the world at large, how great is the ignorance which most people display on his special subject. Hence, unless such a man is imbued with a high sense of duty and believes in the principle of doing the work, and sinking the individual in the work, he may do more harm than good. If the consultant be a man of parts, then he becomes well-nigh indispensable to his day and generation. Among other useful purposes which he is able to fulfil is that of historian of his speciality. He remembers what things were, and is so able to compare and precisely weigh the amount of progress which has taken place in the fields he has set himself to cultivate.

In no branch of the field of knowledge has more progress been made than in that of hospital administration. Our medical institutions have been quietly revolutionised during the last quarter of a century, and the best of them bear little or no resemblance to-day to what they were twenty-five years ago. Indeed, it is satisfactory to record that the administrative advance has not confined itself to one portion of the system, but has extended all along the line. Take, for instance, the type and work of the permanent staff of our hospitals. The secretary of to-day exercises a much wider influence because the best of them display so intelligent an interest in everything which concerns the welfare and progress of the charity represented. Formerly it was held to be wrong to spend money upon many things which are now regarded as essentials in every well-managed institution. The old bad days known as the "good enough" period have practically disappeared so far as this country is concerned, if we except a few scattered institutions far away from the centres of population, or at any rate of culture and advancement. The hospital matron, too, now possesses an influence and education, and displays a vigour and intelligence which were conspicuous by their absence, with a few brilliant exceptions, in 1865. This development was originally due to the work and example of Miss Nightingale, and to the devotion and common sense displayed by Mrs. Wardroper, the then matron of St. Thomas's Hospital. We have recently been inspecting some of the hospitals in various parts of the country, and everywhere found that the matron is in advance of her committee, and that if she had her way the condition of the wards and the surroundings of the patients and of the nursing staff would be even better than they are, despite the great improvement of recent times. The secretaries and matrons who would oppose the erection of a nurses' home, and the provision of a separate bedroom, together with dining and recreation rooms for the nurses, are so rare as to be noteworthy. All this is most encouraging to the public and honourable to the officials, to whom great credit is undoubtedly due.

Then again, another welcome change is the improvement in the quality of the food issued to the patients and the condition in which it is served. Formerly hospital patients too frequently found it difficult to eat what was put before them, not because the food was initially bad, but on account of the unappetising way in which it was served up. This improvement in the service must no doubt be placed to the credit of the sisters and nurses, whose whole-hearted devotion to the sick is beyond all praise. The sister and nurse of to-day feel it to be a reflection upon their honour to allow any food to be placed before a patient which they would have any difficulty in eating themselves. No doubt the secretaries and matrons have represented the matter to the committees, which have taken steps to improve the quality of the food and to insist upon it being properly cooked, still no contractor and no cook can secure the issue of a tempting diet to a sick person unless they have the co-operation and thoughtful care of an intelligent and devoted nurse.

Improved hygiene has also done much to lessen the risks of hospital treatment. Even so recently as ten years ago hardly a hospital had a proper plan of its drainage, and where such existed, it was found, when tested, to be incomplete, misleading, or seriously inaccurate. A gentleman, well known in the hospital world, in 1883 commenced to collect plans of all the existing medical institutions in Great Britain, and afterwards extended his researches to foreign countries. The result of his efforts has been to direct the attention of hospital managers to their system of drainage, and so it has gradually come to pass that more and more institutions have been redrained, and the board-rooms of most well-regulated hospitals now contain a correct plan of their drainage systems. How much this has tended to improve the condition and the atmosphere of the wards it would be difficult to estimate.

So in matters of construction, the expert is able to show by reference to existing buildings exactly the date when each improvement was introduced. Originally, as is still the case in some foreign countries, two, and even four, patients were placed in one bed. Nowadays such a thing when mentioned is disbelieved by the ordinary individual. Not only does each patient have a bed to himself, but that bed is allotted a certain proportion of cubic and superficial space and window surface. Linked beds placed in wards without cross ventilation, and corner beds which were nothing better than nests for the propagation of erysipelas and pyæmia, have largely disappeared. The model ward of to-day has rounded corners, windows so constructed as to secure that each bed shall have its separate wall space, with a window on each side of it. The corner beds have disappeared, whitewashed walls and drab dados have given place to tinted or painted walls and dados of encaustic tiles, whilst pictures, flowers, birds, invalid furniture, and other accessories make the majority of hospital wards pleasant sick chambers instead of dull abodes of suffering and gloom.

It will be noticed that we have placed first the improvement in the class and qualifications of the chief members of the permanent staff in this survey. This we have done because, strange to say, except in the best provincial and some Scotch hospitals, the boards of managers have not progressed with the times. Only a fortnight ago we pointed out that in Canterbury so little interest was taken in the administration of the hospital, that it was seriously proposed to abolish the annual court, and thereby to lessen the opportunity for the exhibition of outside interest and independent co-operation. What gave rise to this proposal? The active managers, who are no doubt very limited in number, felt it to be out of their power to induce a quorum of governors to assemble on these occasions. How many board-rooms exhibit a majority of empty chairs on committee days! This absence of zealous workers in the cause of sickness and suffering is a serious reflection upon our day and generation. It arises in London from the fact that the last thing most men will do is to give a portion of their time to the discharge of the duties of an honorary manager of a public institution. Such men argue that time is money, and as competition increases, the tendency is more and more to silence those who solicit co-operation by producing cheque-book and loosening the purse strings. The younger men feel that all their energies must be employed in making their business a success, or they will be left behind in the race. The older men, so soon as they have made a competency, flee to the country, and spend as little time as possible in the metropolis. No doubt all this is to be regretted, because there never was a time when anything like so much money was given to hospitals. The *Hospital Annual* testifies to this fact beyond all dispute, and the point we wish to urge is that there is something better than money which the hospitals at present want, though of course they still need money. This is the whole-hearted devotion and honorary service of men of capacity and intelligence who, though their means may be small, will still give what they have to the service of the sick. Might we suggest that those hospital governors who would like to take a part in the active management of the institutions to which they subscribe, should intimate this fact to the secretaries of the various institutions? We are of opinion that it is desirable for a governor to first serve as a house visitor before he seeks a seat on the committee of management. The duties of a house visitor enable him to get an insight into the administration of an institution which must make him a relatively better committeeman than one who has not served in this capacity. If hospital governors would kindly remember that their duty to the hospitals does not cease when they have sent their annual contribution, but that good service heartily rendered is without price; then it would be the rule, and not the exception, to find full attendances at the boards, which must result in advantage to everybody concerned. There is no commoner cry nowadays than that of those who complain they do not know what to do with their leisure. The hospitals need the assistance of really capable and intelligent governors, and we hope that many such may be found amongst the subscribers to the Metropolitan hospitals especially, who will take the trouble to intimate to the secretaries their willingness to take a part in the administration. If this can be secured the administrative advance will then leave little, if anything, to be wished for.

